# Long-Term Culturing of FreeStyle 293-F Cells Affects Immunoglobulin G Glycome Composition

**DOI:** 10.3390/biom13081245

**Published:** 2023-08-14

**Authors:** Fran Lukšić, Anika Mijakovac, Goran Josipović, Vedrana Vičić Bočkor, Jasminka Krištić, Ana Cindrić, Martina Vinicki, Filip Rokić, Oliver Vugrek, Gordan Lauc, Vlatka Zoldoš

**Affiliations:** 1 Department of Biology, Faculty of Science, University of Zagreb, 10000 Zagreb, Croatia; 2 Genos Glycoscience Research Laboratory, 10000 Zagreb, Croatia; 3 Laboratory for Advanced Genomics, Division of Molecular Medicine, Ruđer Bošković Institute, 10000 Zagreb, Croatia; 4 Department of Biochemistry and Molecular Biology, Faculty of Pharmacy and Biochemistry, University of Zagreb, 10000 Zagreb, Croatia

**Keywords:** IgG glycosylation, long-term cell culturing, FreeStyle 293-F, IgG glycoengineering, cell aging

## Abstract

Glycosylation of IgG regulates the effector function of this antibody in the immune response. Glycosylated IgG is a potent therapeutic used for both research and clinical purposes. While there is ample research on how different cell culture conditions affect IgG glycosylation, the data are missing on the stability of IgG glycome during long cell passaging, i.e., cell “aging”. To test this, we performed three independent time course experiments in FreeStyle 293-F cells, which secrete IgG with a human-like glycosylation pattern and are frequently used to generate defined IgG glycoforms. During long-term cell culturing, IgG glycome stayed fairly stable except for galactosylation, which appeared extremely variable. Cell transcriptome analysis revealed no correlation in galactosyltransferase *B4GALT1* expression with galactosylation change, but with expression of *EEF1A1* and *SLC38A10*, genes previously associated with IgG galactosylation through GWAS. The FreeStyle 293-F cell-based system for IgG production is a good model for studies of mechanisms underlying IgG glycosylation, but results from the present study point to the utmost importance of the need to control IgG galactosylation in both in vitro and in vivo systems. This is especially important for improving the production of precisely glycosylated IgG for therapeutic purposes, since IgG galactosylation affects the inflammatory potential of IgG.

## 1. Introduction

Glycosylation is a highly versatile post-translational modification that greatly extends the structural and functional flexibility of proteins. The addition of glycans is a complex, multi-step process that can affect the physical and biological properties of proteins [1,2,3,4]. Therefore, the strict control of glycosylation patterns became an obligatory prerequisite for the development of therapeutic proteins. Protein glycoengineering is frequently performed in vivo using different mammalian and non-mammalian cell expression systems to obtain precisely glycosylated proteins [5,6,7]. However, the glycosylation machinery of non-mammalian cells differs from the one found in humans, which results in the expression of recombinant proteins decorated with non-human glycans [7,8,9,10,11]. The same problem occurs with some mammalian cell types, such as the Chinese hamster ovary (CHO) cell line, the most widely used cells for the production of recombinant proteins. CHO cells cannot fully replicate human glycosylation patterns as they lack some key glycosylation enzymes and express proteins with additional glycan residues that can be immunogenic [12,13,14]. Additionally, instability of this cell line during long-term culturing results in a change in phenotype drift and decrease of antibody titer [15]. HEK293 cells are also recognized as a robust protein expression platform with the advantage of producing proteins with human-like glycosylation patterns [16,17,18,19]. Moreover, HEK293 cells were adapted for large-scale biopharmaceutical production of proteins by clonal selection to yield the FreeStyle 293-F suspension cell line with maximized protein production [20,21,22,23]. FreeStyle 293-F cells turned out to be an especially good platform for the production of immunoglobulin G (IgG) with a very similar glycosylation pattern to that present in human IgG [16,24,25,26]. IgG glycoengineering is of major significance because monoclonal antibodies (mAbs) represent the largest set of protein therapeutics that is indicated for a variety of non-communicable diseases [27,28].

IgG is a central glycoprotein in human immunity that consists of the Fab region responsible for binding of antigens and the Fc effector region that interacts with downstream immune elements to elicit an appropriate immune response. Glycans attached to the Fc region serve as key regulators that fine-tune the effector function of IgG antibodies in the immune milieu [29,30,31]. Alternative IgG glycosylation is associated with a plethora of pathological conditions and ample experimental data demonstrate that glycans affect the biological properties of IgG-based therapeutics [32,33]. One of the examples is glycoengineered afucosylated mAbs, with an increased capacity to induce antibody-dependent cellular cytotoxicity (ADCC), which is heavily exploited in cancer treatment [34,35,36]. A similar pro-inflammatory effect is observed in IgGs with increased bisecting *N*-acetyl glucosamine (GlcNAc) [37]. Research on intravenous immunoglobulin (IVIg) therapy revealed that the anti-inflammatory properties of IVIg are mostly fueled by sialylation of IgG [38,39,40]. IgG galactosylation is also related to reduced inflammation, and the absence of galactose from IgG glycans strongly associates with chronic inflammation in disease and aging [41,42]. Even though the significance of IgG glycans in inflammation is well established, the regulatory mechanisms underlying the role of alternative IgG glycosylation are not well understood. This gap undermines the current in vivo glycoengineering endeavors to generate IgG with a defined pattern of glycosylation. The main approaches of in vivo glycoengineering include overexpression or disruption of main enzymes in the general glycan biosynthesis pathways, manipulation of environmental factors and supplementation with different glycan-modifying compounds [33]. All of these approaches rely on the hypothesis that the composition of the IgG glycome in cell culture conditions is stable when no intervention is performed. However, a recent study by Kaur et al. reported that long-term culturing of CHO cells used in the production of mAbs results in a decrease in antibody titer, reduction of host cell protein production, phenotypic drift, perturbation of charge heterogeneity, and changes in the glycan profile in late passages of cells [43].

The aim of this study was to test the stability of IgG glycome composition during cell culturing of FreeStyle 293-F cells, which secrete recombinant IgG with a human-like glycosylation pattern and are frequently used to generate defined IgG glycoforms. For this, we established a controlled time course experiment corresponding to 40 cell passages. We found that IgG glycan traits are quite stable besides IgG galactosylation, showing extremely high variability, suggesting that the number of cell divisions should be controlled if this cell line is used for the production of IgG with defined glycosylation. Considering that the regulatory pathways of IgG glycosylation are mainly unknown, which hinders the precise IgG glycoengineering efforts, we also performed a transcriptome analysis in an attempt to identify some genetic elements responsible for the observed changes of IgG galactosylation. Interestingly, no disturbance in the glycan biosynthesis pathway was observed highlighting the need to deepen the studies of IgG glycosylation regulation to perfect the in vivo glycoengineering endeavors.

## 2. Materials and Methods

### 2.1. Cell Culture and Transfections

The FreeStyle™ 293-F cell line (Gibco, Grand Island, NY, USA) was maintained in FreeStyle™ 293 Expression Medium (Gibco, USA) in 125 mL Erlenmeyer flasks (Nalgene, Rochester, NY, USA). The cells used in all experiments were cultured according to the protocol from Vink et al. [16] under controlled conditions; they were kept in a shaker incubator at 37 °C in a humidified atmosphere with 8% CO_2_ and agitated at 125 rpm. Cells were passaged when the culture reached a density of around 2 × 10^6^ viable cells/mL (every 2–3 days). Population doubling time for this cell line is approximately 24 h according to the manufacturer, meaning the cells were passaged every 2–3 population doublings. The transfection of cells was performed every four passages, starting from passage three. All transfections were performed using 293fectin Transfection Reagent (Gibco, USA) and according to the optimized protocol described in Mijakovac et al. [44]. Briefly, cells were plated in non-treated 6-well plates at a concentration of 500,000 cells/mL and transfected with 2 μg of a plasmid containing IgG heavy and light chain, p3SVLT, pORF-hp21, and pORF-hp27 in the ratio: 0.69/0.01/0.05/0.25 [15]. Cell suspensions were collected 5 days after transfection and centrifuged at 4000× *g* for 5 min. Obtained supernatants were used for IgG isolation and subsequent glycan analysis, while cell pellets were used for gene and protein expression analysis. Three independent time course experiments were performed using described methodology. The only difference between the independent experiments was the passage number of cells when the time course experiment was initiated: 3rd passage for experiment 1; 7th passage for experiment 2; and 11th passage for experiment 3. Duration of experiment 1 was 96 days and IgG glycome was analyzed at 10 time points; duration of experiment 2 was 88 days and IgG glycome was analyzed at 9 time points; duration of experiment 3 was 80 days and IgG glycome was analyzed at 8 time points.

### 2.2. Calculation of Total Cell Age

Total cell age represents the number of passages cells have gone through since the initial thawing of the original cell aliquot. It is calculated as a sum of passages from the initial thawing until freezing and all passages thereafter. Therefore, since the cells used in the first experiment were an aliquot of the original cells frozen at passage 3, their total age at the beginning of the experiment was 3 passages rather than the customary zero passages. Likewise, the cells used in experiments 2 and 3 were aliquots of the original cells frozen at passages 7 and 11, making their total age at the start of the experiment 7 passages and 11 passages, respectively.

### 2.3. Isolation of IgG

IgG was isolated from FreeStyle 293-F cell culture supernatants using the same procedure as previously described [44]. In short, affinity chromatography using 40 µL of Protein G Agarose fast flow bead slurry (Merck, Darmstadt, Germany) was used to capture IgG from 2 mL of cell culture supernatant. IgG was eluted from the beads with 100 µL of 0.1 mol/L formic acid (Merck, Germany) and neutralized with 17 µL of 1 mol/L ammonium bicarbonate (Merck, Germany). Slight modification of the previously described procedure involved the use of the Orochem filter plate (Orochem Technologies Inc., Naperville, IL, USA) as the device on which all washing steps, after incubation of samples with Protein G beads as well as elution of IgG, were performed.

### 2.4. IgG Glycan Preparation

Glycans from IgG protein were prepared according to the previously described protocol [44], which consists of three basic steps: glycan release from the protein, glycan labeling, and purification of glycans. Glycans were enzymatically released from IgG using PNGase F (Promega, Madison, WA, USA); the released IgG glycans were then labeled with a fluorescent dye procainamide (Thermo Fisher Scientific, Walthman, MA, USA) and purified using liquid chromatography solid-phase extraction (HILIC-SPE) on a 0.2 μm wwPTFE filter plate (Pall Corporation, Ann Arbor, MI, USA).

### 2.5. Analysis of IgG Glycome Using HILIC-UPLC Method

Fluorescently labeled IgG glycans were analyzed by hydrophilic interaction liquid chromatography (HILIC) on an Acquity Ultra-Performance Liquid Chromatography (UPLC) instrument (Waters, Milford, MA, USA), as previously described in detail [44]. The obtained UPLC chromatograms were all separated in the same manner into 24 peaks and the amount of glycans in each peak was expressed as a percentage of the total integrated area. The glycan structures corresponding to each individual peak were determined previously [44].

### 2.6. RNA Isolation and RNA Sequencing

Total RNA from cell pellets was extracted using the RNeasy Mini Kit (Qiagen, Hilden, Germany) and treated with TURBO DNase (Invitrogen, Carlsbad, CA, USA) to remove residual DNA. Total RNA collected from the cells at passages 7, 23, 27, and 35 (total cell age after the correction for the passage number) from experiments 1 and 2 were sent to Macrogen (Seoul, Republic of Korea) for RNA sequencing using the NovaSeq 6000 Sequencing System (Illumina Inc., San Diego, CA, USA). Differential expression analysis was performed using the DRAGEN Differential Expression tool (Illumina), which utilizes the DeSeq2 algorithm to identify differentially expressed genes in the two conditions. Each passage was compared to passage 7 and two neighboring passages to identify differentially expressed genes (adjusted *p*-value < 0.01). Finally, the same passages from two experiments were compared to identify genes that changed in the same pattern in both experiments.

### 2.7. Quantitative Real-Time PCR (qPCR)

Reverse transcription after DNase treatment was performed using the PrimeScript Rtase (TaKaRa, Shiga, Japan) and random hexamer primers (Invitrogen, USA). Quantitative real-time PCR was performed using the 7500 Fast Real-Time PCR System with the SYBR Green Gene Expression Assay. Sequences of primers used (5′-3′) were: SLC38A10_FW_2 ATGGATGACGCACCAGTCG, SLC38A10_REV_2-CTTCCCGTAGGCGTGGAATG, EEF1A1_FW_2-TTGGACACGTAGATTCGGGC, EEF1A1_REV_2-CGTTCACGCTCAGCTTTCAG, B4GALT1_FW_3-CCAGGCGGGAGACACTATATT, B4GALT1_REV_3-CACCTGTACGCATTATGGTCAT, GUSB_FW1-CCACGGTGTCAACAAGCAT, GUSB_REV1-GCGGGGGACCGGGAA. The mean value of five replicates was normalized to GUSB expression, which was used as an endogenous control. Fold changes in transcript expression were calculated compared to the first time point of every experiment using the ΔΔCt method [45].

### 2.8. Protein Extraction and Western Blot Analysis

For western blot analysis, the cells were lysed in RIPA buffer (50 mM TRIS pH 7.5; 0.1% Triton X, 1 mM EDTA, 135 mM NaCl) supplemented with Protease Inhibitor Cocktail (cOmplete™ ULTRA Tablets, EDTA-free, glass vials Protease Inhibitor Cocktail, Roche, Switzerland). Three replicates of isolated proteins were pooled together and 20 μg of proteins in total was separated on 10% polyacrylamide gels and transferred to nitrocellulose membranes (Amersham™Protran^®^ Premium 0.45μm NC; GE Healthcare, Chicago, IL, USA). The membranes were blocked for 1 h with TBS-T containing 5% milk (Milchpulver blotting grade, Roth, Karlsruhe, Germany) prior to incubation with primary antibodies. The primary antibodies used were diluted in blocking buffer as follows: 1:1000 anti-B4GALT1 (ab121362, Abcam, Cambridge, UK), anti-β actin 1:1000 (sc-69879, Santa Cruz Biotechnology, Santa Cruz, CA, USA). Incubations were performed overnight at +4 °C. The membranes were then washed and incubated in HRP conjugated secondary goat anti-mouse or goat anti-rabbit antibodies (ab205719 and ab6721, respectively, Abcam) for 1 h at room temperature. The signals were developed using Immobilon Western Chemiluminescent HRP Substrate (Merck, Germany) and photographed using the Alliance Q9 Advanced imaging system (Uvitec, Cambridge, UK). The protein signals were quantified using ImageJ [46].

### 2.9. Statistical Analysis

Derived glycan traits were calculated as a sum of all measured glycan peaks with the same characteristic (galactosylation, agalactosylation, sialylation, core fucosylation, and bisecting GlcNAc). A list of all individual glycan peaks used to calculate derived traits is included in the Table S1. The statistical significance of glycan and expression changes was calculated using R [47]. The unpaired two-tailed Student’s *t*-test was used to determine the statistical significance of changes between passages and results with an adjusted *p*-value < 0.05 were considered statistically significant. Principal component analysis (PCA) plot was generated using the factoextra and FactoMineR packages in R with gene expression values.

## 3. Results

### 3.1. IgG Galactosylation Changes Extensively during Cell Culturing

**Figure 1:**
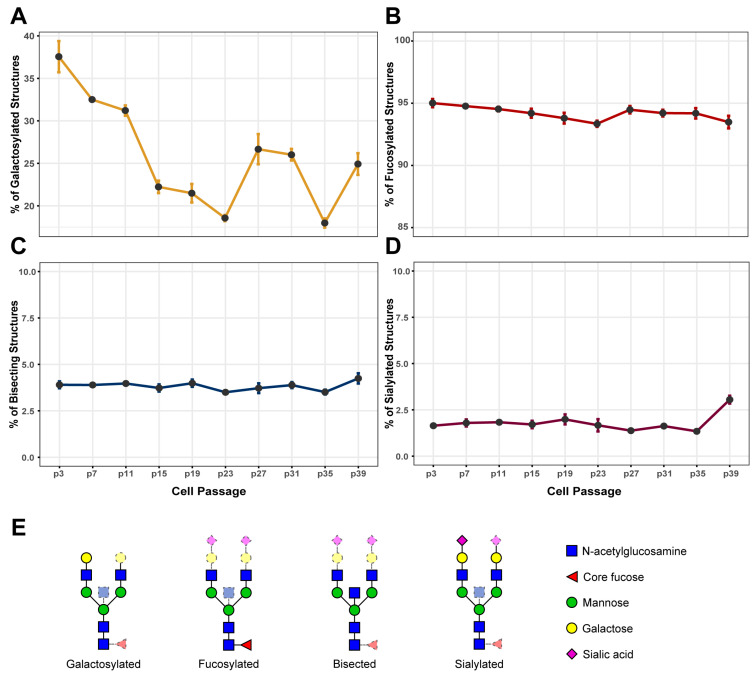
Changes of derived immunoglobulin G (IgG) glycan traits in FreeStyle 293-F cells during the course of 40 passages (time course experiment 1). (**A**) IgG galactosylation; (**B**) IgG core fucosylation; (**C**) IgG bisection; (**D**) IgG sialylation; (**E**) graphical representation of the derived IgG glycan traits [48]. Statistical data are not included in the figure to simplify the display and can be found in Tables S3–S6.

In the time course experiment, the FreeStyle 293-F cells were passaged 40 times before their viability fell below 90%, which is considered the end of the log phase of growth based on the manufacturer’s user manual. In total, cells were transfected with IgG expression plasmid at 10 time points, each 9 or 10 days apart. By analyzing the total IgG glycome, we confirmed that the glycosylation pattern of IgG secreted from FreeStyle 293-F cells resembled the glycosylation pattern of IgG from human plasma [16,24,44]. Almost all IgG glycans were core fucosylated, around 18–38% were galactosylated, and only a small proportion were sialylated or contained bisecting GlcNAc. Galactosylation appeared to be the most variable IgG glycan trait during prolonged passaging of the FreeStyle 293-F cells: the percentage oscillated from 18–38% depending on the passage number (Figure 1). During the first 23 passages, the relative amount of galactosylated IgG was steadily decreasing, but in later passages the pattern of change started to fluctuate. There was a significant increase of IgG galactosylation in passage 27, no change of galactosylation in passage 31, a significant decrease in passage 35, and another significant increase in passage 39 (Figure 1, Table S2).

At the level of individual glycan structures, monogalactosylated core fucosylated (FA2(6)G1 (GP8) and FA2(3)G1 (GP9)) IgG glycoforms were the two most abundant galactosylated IgG glycoforms secreted from FreeStyle 293-F cells (Figure S1). Together, they accounted for more than a quarter of all IgG glycoforms and their change explains most of the IgG glycome variability observed in FreeStyle 293-F cells (Figure S1, Tables S8 and S9). Digalactosylated core fucosylated (FA2G2 (GP14)) IgG glycoforms accounted for the residual IgG galactosylation variability (Figure S1, Table S10). Notably, the direction of change for monogalactosylated (G1) and digalactosylated (G2) IgG glycans was the same between all measured time points. Among the agalactosylated IgG glycans, the most abundant was the agalactosylated core fucosylated glycan (FA2 (GP4)) present in 50–70% of all IgG glycans (Figure S1, Table S7).

Unlike IgG galactosylation, other derived glycan traits—core fucosylation, sialylation, and bisecting GlcNAc—remained quite stable during cell passaging (Figure 1). In cells from early passages, around 95% of all IgG glycoforms were core fucosylated and, while there was a minor decrease to around 93% in passage 23, core fucosylation was mainly stable. Only around 3–4% of the IgG glycan pool from FreeStyle 293-F cells contained bisecting GlcNAc, and there were no significant changes of this trait through cell passaging (Figure 1, Table S5). Sialylation is the least frequent modification of IgG secreted from FreeStyle 293-F cells (Figure 1). In total, less than 2% of all IgG glycans were sialylated and there were almost no disialylated IgG glycans (Figure 1). A significant change in IgG sialylation was observed in passages 31, 35, and 39 (Table S6). However, the low abundance of IgG sialylation complicated the glycan analysis workflow, as the low intensity of sialylated glycan peaks in obtained chromatograms made it hard to measure them accurately.

### 3.2. The Variability of IgG Glycome Composition during Cell Culturing Replicates between Independent Experiments

**Figure 2:**
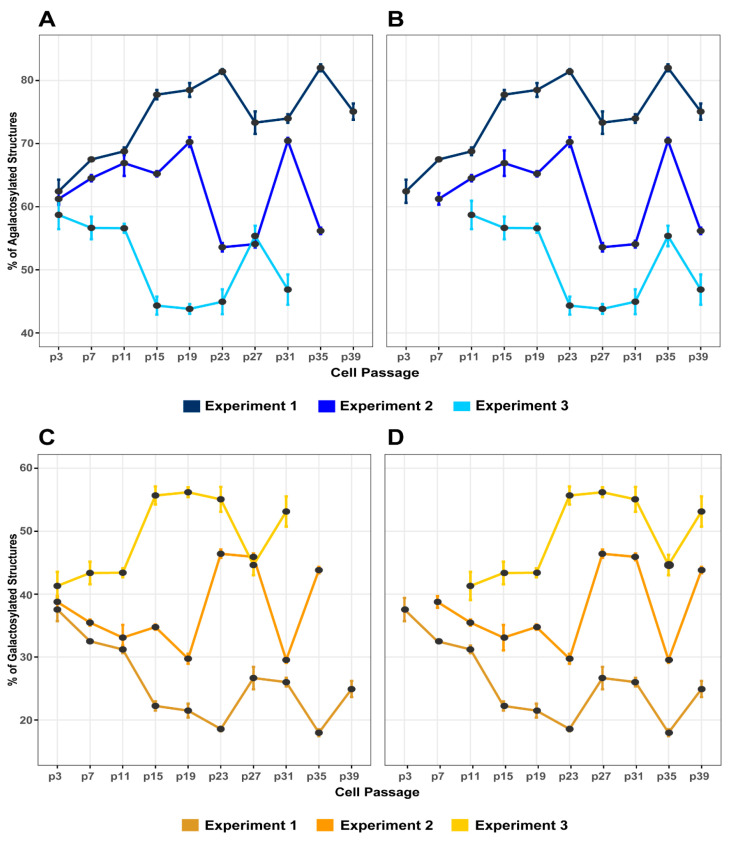
Changes of IgG galactosylation in FreeStyle 293-F cells from the three independent time course experiments. IgG agalactosylation (**A**) before and (**B**) after the correction for passage number. IgG galactosylation (**C**) before and (**D**) after the correction for passage number. Statistical data are not included in the figure to simplify the display and can be found in Tables S2 and S3.

To investigate if the observed changes of IgG glycome composition were a consequence of cell passaging or some inadequately controlled cell culturing condition, the experiment was replicated twice. In time course experiment 2, the cells were passaged 38 times before their viability fell below 90%. In total, 9 transfections were performed in a period of 90 days. Again, galactosylation was the most variable trait that changed significantly in both directions depending on the passage number (Figure 2, Table S2). Other traits—core fucosylation, bisecting GlcNAc, and sialylation—remained almost completely stable throughout the whole time course experiment (Figure S2, Tables S4–S6). Around 90–95% of all glycans from secreted IgG were core fucosylated, similar to what was observed in experiment 1. Although the percentage of IgG glycans with bisecting GlcNAc remained stable with time, the total amount of glycans with bisecting GlcNAc was somewhat higher than in experiment 1 (Figure S2). IgG sialylation remained stable at around 2%, as was observed in experiment 1, and changed significantly only in the last time point.

While comparing the changes of galactosylation in both experiments, a similar trend of change was observed depending on the passage number of cells. However, the major change concerning IgG galactosylation occurred one time point earlier in experiment 2 when compared to experiment 1 (Figure 2). The cells from experiment 1 were an aliquot of the original batch frozen at the third passage, while cells in experiment 2 were an aliquot of the original batch frozen at the seventh passage. When we corrected the data for the total passage number (total age of the cells), the diagram of galactosylation changes revealed almost identical patterns for both experiments (Figure 2). During the first 23 passages (when corrected for the total cell age), there was a significant decrease in galactosylated IgG glycans, but in passage 27 a major increase in galactosylated IgG glycans was observed. Another significant decrease in the percentage of galactosylated IgG glycans occurred between passages 31 and 35, followed by an increase in passage 39. The percentage of agalactosylated glycans changed in the opposite direction of galactosylation, so the pattern was also identical when normalized to the total age/passage number (Figure 2). Other glycan traits remained quite stable throughout the time course experiment, with a slight increase of IgG sialylation at the last time point (Figure S2).

Time course experiment 3 started with an aliquot of the original batch frozen at the 11th passage. Without the correction for the total passage number, the IgG galactosylation pattern seemed quite different from that in time course experiments 1 and 2 (Figure 2). However, after the correction for the total passage number, it became very similar to the one observed in the first two time course experiments with some differences (Figure 2). No decrease of IgG galactosylation was observed at the first few time points and a major increase in IgG galactosylation occurred one-time point earlier than in the first two time course experiments (Figure 2). These slight differences between the independent experiments can be explained by the fact that the number of cell passages did not reflect the accurate number of cell divisions.

### 3.3. Genetic Background of IgG Glycosylation Changes

In our effort to map the regulatory mechanisms responsible for the observed changes of IgG galactosylation during cell culturing, we performed analysis of the cell transcriptome. Based on the extent of glycan changes, four time points were chosen for RNA sequencing: passages 7, 23, 27, and 35 from experiment 1 and 2. The passages from experiment 2 were corrected for the total cell age prior to the analysis. We observed a clear grouping of the samples, which was in concordance with the glycan changes (grouping of passages 7 and 27, and grouping of passages 23 and 35; Figure S3). When analyzing the expression of individual genes, we observed that many of them changed in the same direction as IgG galactosylation. A list of the most significant 10 genes whose expression followed the pattern of IgG galactosylation change is shown in Table 1.

**Figure 3:**
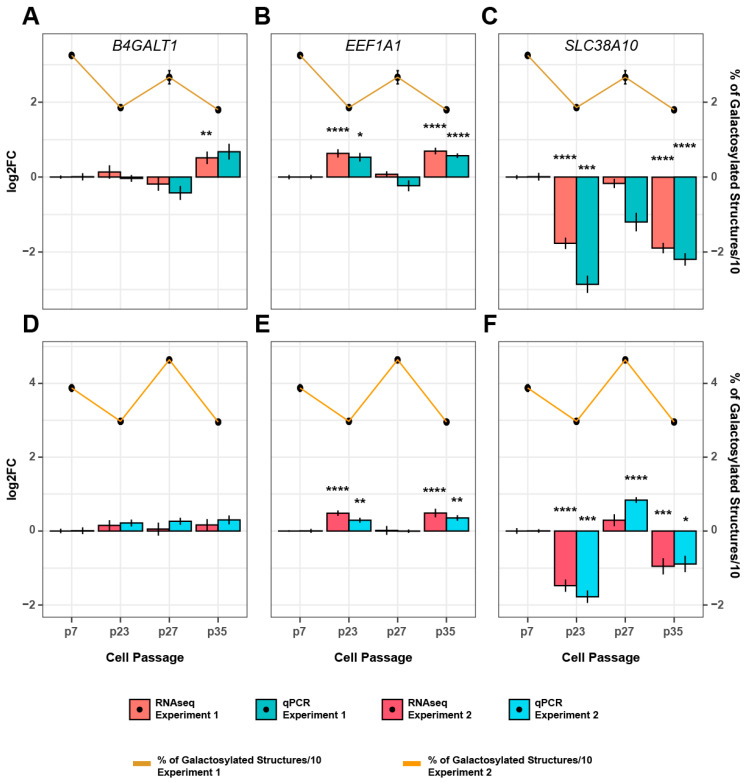
Correlation of *B4GALT1*, *EEF1A1,* and *SLC38A10* expression and IgG galactosylation. Changes in the expression of the *B4GALT1* (**A**,**D**), *EEF1A1* (**B**,**E**), and *SLC38A10* (**C**,**F**) genes in correlation with the galactosylation changes in experiment 1 (top panels) and 2 (bottom panels), respectively. Transcriptional changes obtained by RNA sequencing were validated by RT-qPCR. Expression levels are shown as fold change relative to gene expression in passage 7 for both experiments. Fold change values are represented in the log2 scale, while the percentage of galactosylated IgG glycans is represented as calculated percentage/10. The *GUSB* gene was used as an endogenous control for qPCR analysis. * *p* adj < 0.05, ** *p* adj < 0.01, *** *p* adj < 0.001, **** *p* adj < 0.0001.

Listed genes from the transcriptome analysis have different functions in the cell, from cellular respiration and cholesterol synthesis to intracellular transport and signal transduction, but none of them are involved in the process of IgG glycosylation. Therefore, we searched the transcriptomic data for the enzymes with known functions in IgG glycosylation, especially galactosylation. Remarkably, none of the glycosylation-related genes could be linked to IgG glycome changes observed in the time course experiments. Moreover, a key glycosyltransferase, B4GALT1 (β-1,4-galactosyltransferase 1), which catalyzes the addition of galactose, had a completely different pattern of expression in two analyzed experiments from the pattern of IgG galactosylation (Figure 3). This was in line with previous genome-wide association studies (GWAS) of IgG glycosylation, which discovered that many genetic loci that associate with IgG glycosylation have no known role in the process of glycosylation [49,50,51,52,53]. So, we integrated the data from different GWA studies of IgG glycosylation and focused on changes of expression in IgG glycome GWAS hits in our two time course experiments. Remarkably, expression of two IgG galactosylation-related GWAS hits, *EEF1A1* (eukaryotic translation elongation factor 1 alpha 1) and *SLC38A10* (solute carrier family 38 member 10), correlated with changes in IgG galactosylation at every time point analyzed. More precisely, *EEF1A1* was upregulated between passages 7 and 23 and passages 27 and 35, and downregulated between passages 23 and 27. In other words, the expression of *EEF1A1* was negatively correlated with IgG galactosylation in both experiments (Figure 3). On the other hand, the expression level of *SLC38A10* was positively correlated with IgG galactosylation (Figure 3). To verify the significance of changes observed in the transcriptome data, we performed additional qPCR analysis on more biological replicates collected at the same time points as the samples for the transcriptome analysis. The results of qPCR verified the expression changes of *EEF1A1*, *SLC38A10,* and *B4GALT1* observed in the transcriptome analysis (Figure 3).

**Figure 4:**
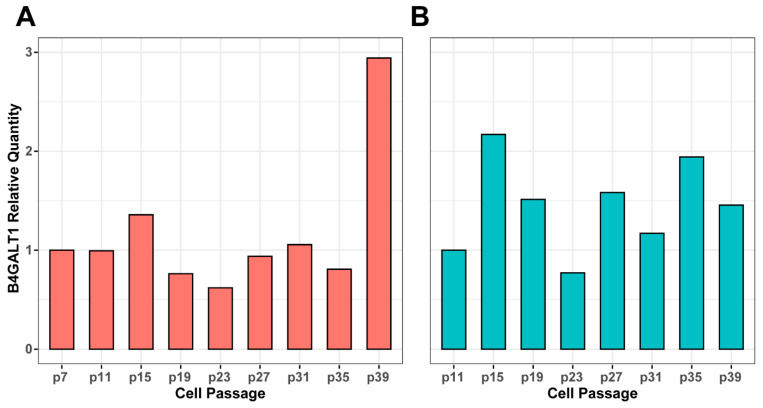
Expression data for *B4GALT1* obtained by the western blot analysis. Relative expression of *B4GALT1* in the protein levels during (**A**) experiment 2 and (**B**) experiment 3 were calculated using densitometry and all changes were normalized to passage 7 in the second or passage 11 in the third experiment.

In order to reveal whether *B4GALT1* expression on the protein level was possibly responsible for the observed changes of IgG galactosylation, we performed western blot analysis on samples from experiments 2 and 3 (Figure S4). We then calculated relative *B4GALT1* protein levels in the individual passages using densitometry analysis and normalized it to the seventh passage to be able to correlate these changes with transcriptional and glycan (galactosylation) changes. While there were no significant transcriptional (mRNA level) changes of *B4GALT1* at the time points analyzed, the protein levels were changing during prolonged passaging (Figure 4). The direction of changes in *B4GALT1* expression on the protein level could not be correlated with the change of IgG galactosylation at most of the time points analyzed. Only protein changes in passages 23 and 39 (0.62-fold decrease in passage 23 and a 2.94-fold increase in passage 39) from time course experiment 2 were in line with the observed glycan changes. These results suggest that IgG galactosylation changed independently from *B4GALT1* expression.

## 4. Discussion

IgG glycosylation is highly variable in human populations and is affected by both genetic and environmental factors. For example, the level of agalactosylated, core fucosylated IgG glycans can vary from 6–50% among healthy people, where galactosylation is the most heterogeneous IgG glycan trait [54,55]. Moreover, the decrease of galactosylated IgG glycoforms accompanies healthy aging and is associated with various multifactorial diseases [41,42]. The variability of the IgG glycome in the population level raises a question about the stability of glycans present in IgG produced by cells in culture. While there is ample experimental data on how different cell culture conditions such as temperature, pH, media additives, cell viability, and other factors greatly affect the glycosylation pattern of IgG, the data are missing on the stability of the IgG glycome during long cell passaging, i.e., cell “aging” [56,57,58,59,60,61]. Instability in mAbs-producing CHO cell lines during long-term culturing has been reported previously, mainly affecting the yield of antibodies produced, and is associated with lower cumulative cell time values and increased sensitivity to cellular stress [15]. Recently, Kaur et al. have reported changes in the glycan profile of IgG produced in CHO cells, resulting in a decrease of fucosylated and an increase of galactosylated structures in IgG produced in late passages of cells [43].

Therefore, we monitored the glycosylation pattern of IgG secreted from the FreeStyle 293-F cell line, which was optimized to produce IgG decorated with similar glycans as IgG from human plasma. We cultured FreeStyle 293-F cells until their viability dropped below 90% (for approximately 100 days) and analyzed the IgG glycan phenotype at 10 time points (every 9 or 10 days). IgG galactosylation proved to be extremely variable, ranging from an average of 18–38% of the total IgG glycome. Other IgG glycan traits remained stable with slight fluctuation of IgG fucosylation and sialylation. The extensive changes of IgG galactosylation in FreeStyle 293-F cells that we have observed raised a major concern about the efficacy of this cell line for the continuous production of IgG with defined glycosylation. Vink et al. (2014) stated that the glycosylation profile of antibodies produced by FreeStyle 293-F cells is highly reproducible, a claim we could not verify [16]. Moreover, Dekkers et al. (2016) used the same cell line system for precise glycoengineering of IgG antibodies and reported high variability of IgG galactosylation and moderate variability of IgG fucosylation, but failed to discuss those observations [24]. It may be likely that the variability they observed was a direct consequence of IgG isolation at different time points of the FreeStyle 293-F cell culture maintenance process. We also utilized this cell line to study the regulation of IgG glycosylation in the past. However, for every performed experiment, we included a control of the same passage number to circumvent the issue of IgG galactosylation variability in FreeStyle 293-F cells.

Different studies have demonstrated that the increase of galactose-containing glycans often leads to an increase in sialylation, a process mediated by ST6GAL1 (ST6 beta-galactoside alpha-2,6-sialyltransferase 1) which can only sialylate already galactosylated IgG glycans [44,62,63,64,65]. In our previous study, we upregulated the *B4GALT1* gene using transient expression system based on FreeStyle 293-F cells, and this intervention resulted in an increase of galactosylated IgG glycans accompanied by an increase of sialylated IgG glycans [44]. In contrast, in the current study, the time-dependent changes of IgG galactosylation were not accompanied by corresponding changes in IgG sialylation. These results suggest the existence of two different mechanisms possibly involved in the regulation of IgG galactosylation, one that affects both the addition of galactose and sialic acid, and another one that affects only the galactosylation of IgG.

During human aging, IgG galactosylation steadily decreases with advancing age [63]. We have observed a similar pattern in our time course experiment up until the 7th time point (approximately after 70 days of cells growing in culture). Previous population studies of plasma-derived the IgG glycome revealed that digalactosylated (G2) IgG glycans robustly decrease with advancing age. On the other hand, the direction of change for monogalactosylated (G1) IgG glycans depends on the precise IgG glycan structure analyzed. [41]. Interestingly, in the time course experiments performed in this study, the steady decrease of IgG galactosylation was reflected in a decrease of both G1 and G2 glycan structures. We have previously reported a simultaneous decrease in G1 and G2 structures after upregulation of a galactosylation-associated gene, *RUNX3* [50,66]. At the same time, we found that the upregulation of another galactosylation-associated gene, *SPINK4*, led only to a decrease of G1 structures in the IgG glycome [65]. A different pattern of change for G1 and G2 glycans was also observed in consecutive studies performed in our lab [44,53,65]. Taken together, these results suggest that different regulatory mechanisms control IgG mono- and digalactosylation. These findings seems to be strengthened by the fact that some diseases are associated with both G1 and G2 IgG glycans and some only with one type and not the other [32].

The steady decrease of IgG galactosylation was suddenly reversed at time point seven (passage 27) in the time course experiments, and was followed by a fluctuating pattern of change until the cell viability dropped below 90%. We hypothesized that the steady decrease of IgG galactosylation in the first 60 days was a consequence of the prolonged culturing of cells, which ultimately resulted in genomic instability and epigenetic deregulation, leading to the erratic changes in the quantity of galactosylated structures observed at the last four time points. In order to rule out this and the possibility that different environmental variances were inadvertently introduced during cell handling, we replicated the time course experiment twice, starting with the cells of different passage numbers. In the replicated experiments, performed one by one to avoid false-replication due to environmental effects, cells were cultured using different lots of growth medium, but all other culturing conditions were kept the same. While the levels of IgG glycans with bisecting GlcNAc and core fucosylation remained constant, with slight change of sialylated IgG glycans at some time points, we observed a very similar pattern of intensive IgG galactosylation change in both replicated time course experiments. This observation suggested a non-random mechanism as a cause for IgG galactosylation changes, although the possibility of unintentional environmental perturbation brought up by cell handling cannot be completely ruled out. The hypothesis about cell deregulation as a cause of galactosylation variability at the last time points in the time course experiments was rejected as well, considering that the reproducibility of the galactosylation pattern was the highest in the late phases of culturing. Interestingly, even though the pattern of change was similar for all three experiments, the level of total galactosylated IgG glycans was increased in every consecutive experiment, with the relative percentage of galactosylated IgG ranging from 18–58% of the total IgG glycome. The magnitude of observed changes raised a concern about the capacity of the FreeStyle 293-F cells to produce stable and defined IgG glycoforms. This finding is especially important in light of recent studies showing that galactose attached to IgG glycans can have a strong inflammatory potential, emphasizing the need to tightly control IgG galactosylation in both in vitro and in vivo systems [67,68,69,70,71]. On the other hand, previous studies demonstrated that the FreeStyle 293-F cell line is a good model with which to study the mechanisms underlying IgG glycosylation if appropriate controls are included in the experiments [44,53,65]. Considering that IgG galactosylation is the most variable trait in humans, the observation that the same is true in FreeStyle 293-F cells can only reinforce the notion that these cells can serve as an adequate model for specific studies of IgG glycosylation.

The similar pattern of change in three independent time course experiments suggested that the mechanisms responsible for IgG galactosylation variability during continuous culturing of FreeStyle 293-F cells are not random. To map possible genetic elements involved in cellular processes that mediated these changes, a transcriptome analysis was performed at four time points where the extent of change was the highest. In line with our previous results and results from others, which demonstrate that the expression of enzymes that synthesize glycans is not the key regulatory mechanism [72,73], no enrichment of any glycosylation-related pathway was observed. This is also in line with numerous GWA studies of IgG glycosylation traits which demonstrate that most of the GWAS hits have no apparent role in the glycosylation process itself [49,50,51,52,53,74]. In our time course experiments, there was no change in *B4GALT1* expression despite the extensive changes in IgG galactosylation. Moreover, at some time points, *B4GALT1* transcription level was negatively correlated with the change of IgG galactosylation. The same was observed on the protein level, ruling out the possibility that the *B4GALT1* expression is regulated post-translationally. On the other hand, we confirmed in our previous study that B4GALT1 catalyzes the addition of galactose to IgG in the FreeStyle 293-F cell line. Up- or down-regulation of *B4GALT1* resulted in extensive change of IgG galactosylation [44]. Again, this suggests the existence of two different mechanisms that are possibly involved in the complex regulation of IgG galactosylation—the B4GALT1-dependent and another, still unknown mechanism that affects IgG galactosylation indirectly.

To further map the genetic factors responsible for the observed changes in IgG galactosylation, we integrated all the data from previous GWA studies [74] and derived all the prioritized genes associated with IgG glycosylation. We compared the GWAS-prioritized genes with the transcriptome data from the time course experiments. Interestingly, the expression of two galactosylation-specific GWAS hits [53], *EEF1A1* and *SLC38A10*, changed significantly at the analyzed time points. At every time point when IgG galactosylation decreased, the expression of *EEF1A1* increased and vice versa. The opposite was true for *SLC38A10,* whose expression was positively correlated with IgG galactosylation changes. We recently manipulated the expression of both genes using CRISPR tools to functionally validate their role in IgG galactosylation in the FreeStyle 293-F cells [53]. dCas9-VPR mediated upregulation of *SLC38A10* resulted in no change of IgG glycosylation, while the upregulation of *EEF1A1* led to a decrease of galactosylated IgG glycans accompanied with an increase of agalactosylated IgG glycans, the same phenotype we observed in this study. Considering that EEF1A1 is one of the most abundant cell proteins involved in an array of different processes, most notably in translation, it is difficult to hypothesize how exactly it affects IgG galactosylation [75,76]. Some previous studies did demonstrate that EEF1A1 can serve as a signal transducer during inflammation, where it can regulate inflammatory cytokines such as interleukin 6, which can affect IgG glycosylation [75,77]. Different studies also show that EEF1A1 has many different non-canonical functions and regulation of IgG galactosylation might be one of them [75,78].

The shifts in the level of galactosylated IgG glycoforms seem to be a result of non-random cellular mechanisms brought up by long passaging of cells in culture. Currently, it is not possible to hypothesize about the exact mechanisms responsible for the instability of IgG galactosylation. Remarkably, these mechanisms are not dependent on the expression of *B4GALT1* and do not involve any classical glycosylation-related gene. However, the IgG galactosylation changes correlated at all measured time points with the transcriptional expression of two GWAS hits for IgG galactosylation, *EEF1A1* and *SLC38A10*. More research on this subject is imperative to elucidate the mechanisms leading to alternative IgG glycosylation in aging and disease, but also to improve the production of precisely glycosylated IgG for research and therapeutic purposes. The discovery that galactosylation of FreeStyle 293-F-secreted IgG is extremely variable together with the fact that IgG galactosylation is very heterogeneous in human populations raises a concern about the stability of this trait in other cell systems. This should be examined in the future, especially in light of recent functional studies, since they demonstrate that galactosylated IgG can have a strong pro-inflammatory potential, which further highlights the need to precisely control this IgG glycosylation trait.

## Figures and Tables

**Table 1:** Top 10 differentially expressed genes from time course experiments 1 and 2 whose expression followed the IgG galactosylation changes in every analyzed time point (passages 7, 23, 27, and 35). The expression level of each gene is shown as the fold change between the analyzed passages; *p* adj < 0.0001.

	Experiment 1			Experiment 2		
Gene	7–23	23–27	27–35	7–23	23–27	27–35
*SAT1*	3.789	0.558	1.397	3.432	0.194	4.102
*INSIG1*	2.683	0.391	2.455	2.352	0.365	2.253
*PIM1*	3.354	0.434	1.727	2.627	0.325	2.389
*CRYAB*	10.416	0.190	2.582	6.680	0.104	5.267
*GOLT1B*	2.937	0.390	1.771	2.173	0.477	1.843
*FUS*	0.405	1.792	0.430	0.440	2.487	0.555
*LINC00342*	0.155	3.137	0.237	0.355	2.437	0.427
*APPAT*	0.204	2.907	0.231	0.281	3.986	0.295
*PLAU*	3.276	0.422	1.560	2.554	0.271	2.251
*MT-CYB*	0.230	3.167	0.185	0.417	2.555	0.480

## Data Availability

The data presented in this study are available in Supplementary Material.

## Supplementary Materials

The following supporting information can be downloaded at: https://www.mdpi.com/article/10.3390/biom13081245/s1. Figure S1: Changes of individual IgG glycan structures in the time course experiment performed in the FreeStyle 293-F cell line; Figure S2: Changes of IgG core fucosylation, bisection and sialylation in three independent time course experiments performed in the FreeStyle 293-F cell line; Figure S3: Principal component analysis (PCA) of the RNA-seq dataset from the time course experiments 1 and 2 showing the splitting of samples by the first two principal axes; Figure S4: western blot assay of *B4GALT1* expression in experiments 2 (A) and 3 (B); Table S1. Derived IgG glycan traits; Table S2. Statistical analysis of the changes in IgG galactosylation between passages in the time course experiments; Table S3. Statistical analysis of the changes in IgG agalactosylation between passages in the time course experiments; Table S4. Statistical analysis of the changes in IgG core fucosylation between passages in the time course experiments; Table S5. Statistical analysis of the changes in IgG bisection between passages in the time course experiments; Table S6. Statistical analysis of the changes in IgG sialylation between passages in the time course experiments; Table S7. Statistical analysis of the changes in GP4 between passages in the time course experiments; Table S8. Statistical analysis of the changes in GP8 between passages in the time course experiments; Table S9. Statistical analysis of the changes in GP9 between passages in the time course experiments; Table S10. Statistical analysis of the changes in GP14 between passages in the time course experiments.
