# Supplementary material for: Long-Term Culturing of FreeStyle 293-F Cells Affects Immunoglobulin G Glycome Composition
Source: Biomolecules. 2023 Aug 14;13(8):1245. doi: 10.3390/biom13081245 (PMC10452533; doi:10.3390/biom13081245)
Supplement: Supplementary file 1 [file biomolecules-13-01245-s001.zip › biomolecules-2532766-supplementary-figures-revised.pdf]

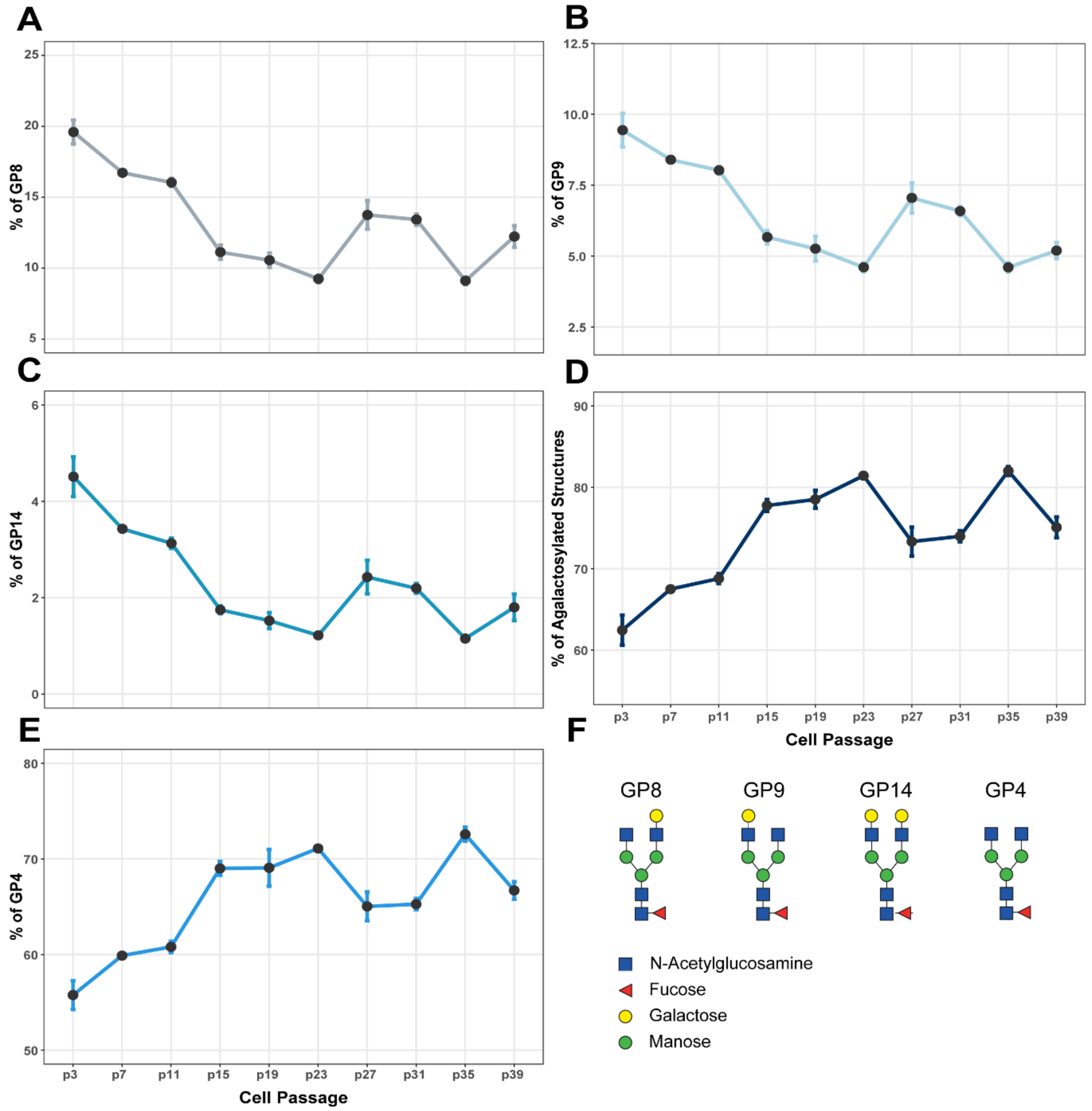

Figure S1. Changes of individual IgG glycan structures in the time-course experiments performed in the FreeStyle 293-F cell line. A) GP8 - monogalactosylated biantennary glycan with 1-6 linkages with core fucose. **B)** GP9 - monogalactosylated biantennary glycan with 1-3 linkages with core fucose. **C)** GP14 - digalactosylated biantennary glycan with core fucose **D)** Agalactosylated IgG glycans E) GP4 - biantennary glycan with core fucose **F)** Depiction of the most abundant glycan structures found on IgG secreted from the FreeStyle 293-F cell line. Note that statistical data is not included in the figure to simplify the display and can be found in Tables S2, S7, S8, S9 and S10.

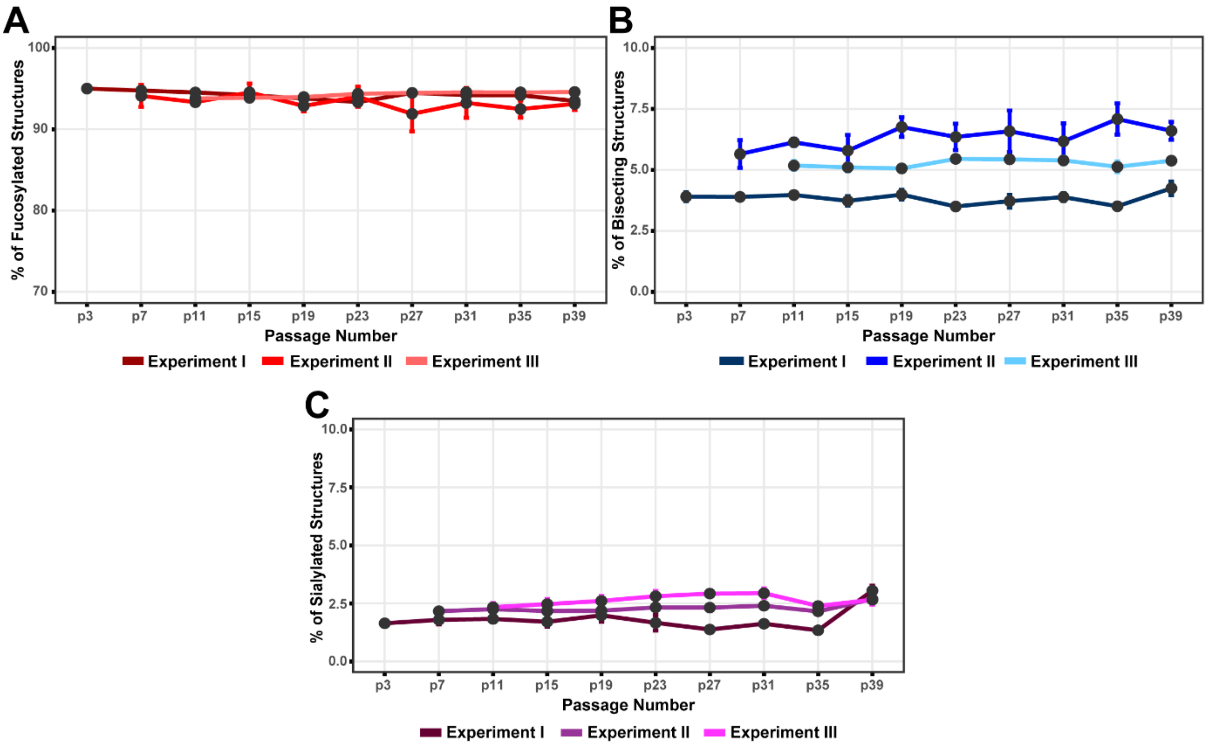

Figure S2. Changes of IgG core-fucosylation, bisection and sialylation in three independent time-course experiments performed in the FreeStyle 293-F cell line. IgG core-fucosylation (**A**), bisection **(B)** and sialylation **(C)** after the correction for passage number. Note that statistical data is not included in the figure to simplify the display and can be found in Tables S4, S5 and S6.

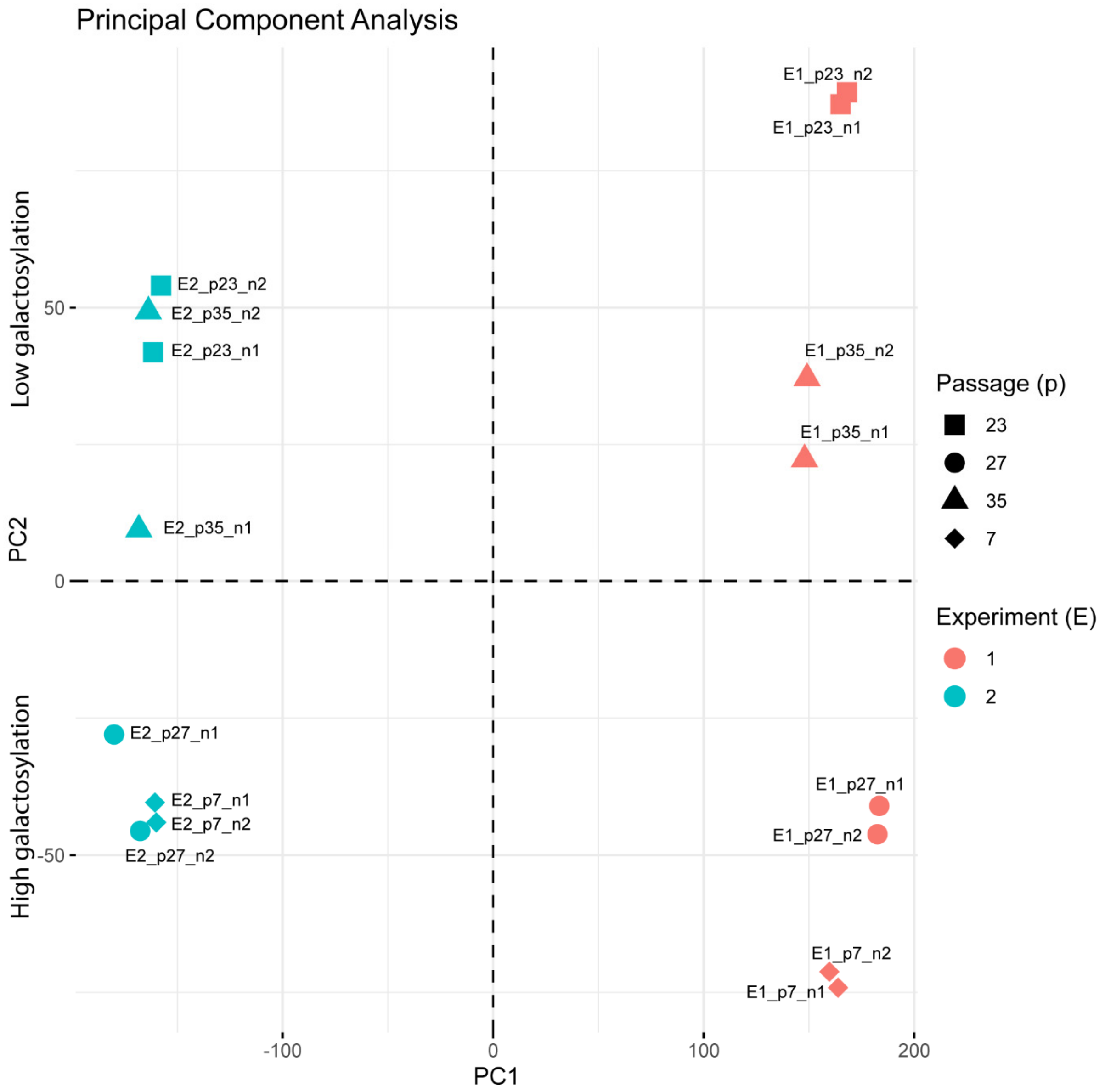

Figure S3. Principal component analysis (PCA) of the RNA-seq dataset from the experiments 1 and 2 showing the splitting of samples by the first two principal axes. Axis 1 corresponds to difference between experiments, while axis 2 corresponds to observed galactosylation changes.

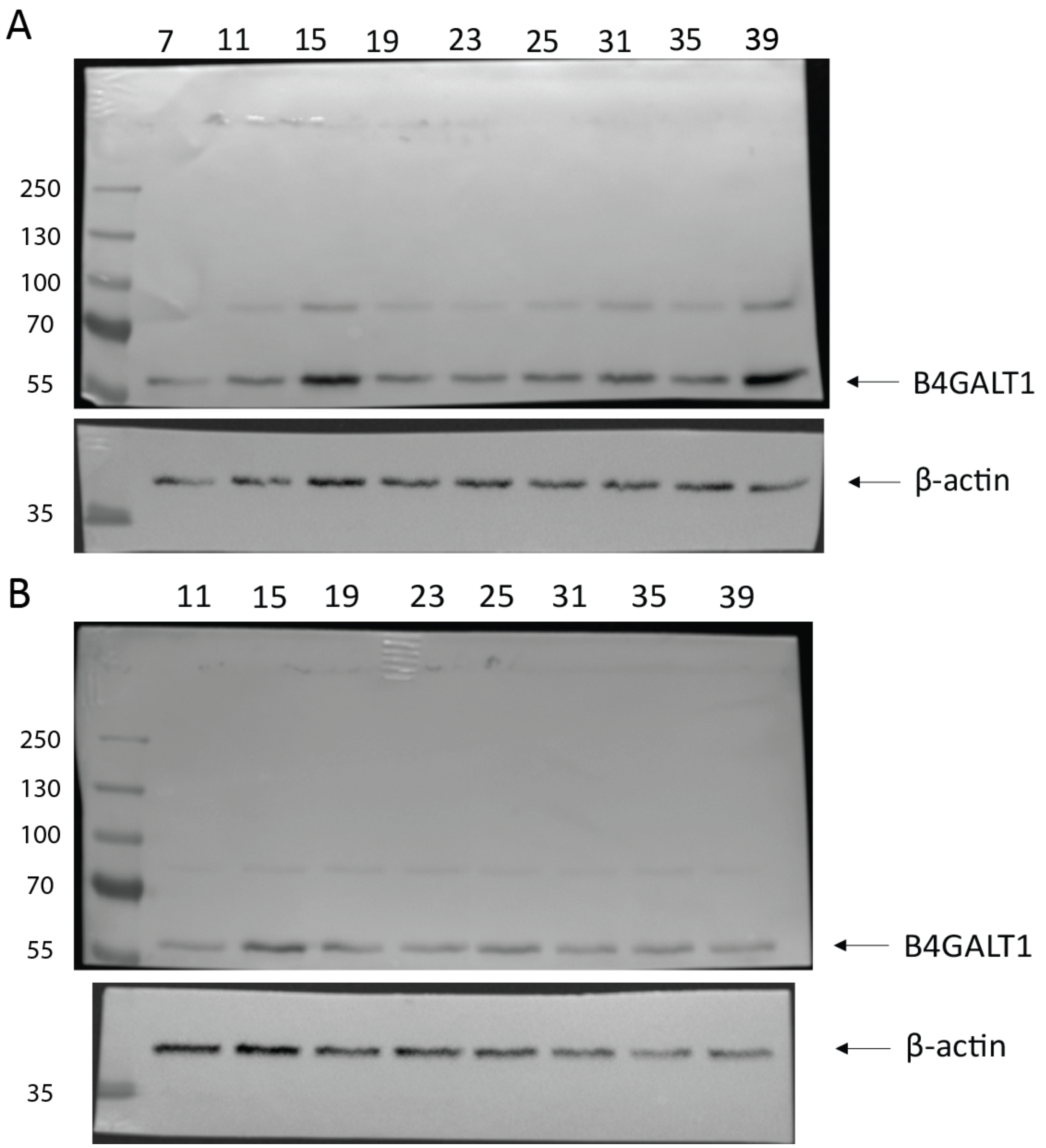

Figure S4. Western blot assay of B4GALT1 expression in experiments 2 (A) and 3 (B). Numbers above indicate passage number. β-actin was used as endogenous control and marker used was PageRuler™ Plus Prestained Protein Ladder, 10 to 250 kDa (Thermo Fisher Scientific, USA).
